# Calreticulin-mutant proteins induce megakaryocytic signaling to transform hematopoietic cells and undergo accelerated degradation and Golgi-mediated secretion

**DOI:** 10.1186/s13045-016-0275-0

**Published:** 2016-05-13

**Authors:** Lijuan Han, Claudia Schubert, Johanna Köhler, Mirle Schemionek, Susanne Isfort, Tim H. Brümmendorf, Steffen Koschmieder, Nicolas Chatain

**Affiliations:** Department of Hematology, Oncology, Hemostaseology, and Stem Cell Transplantation, Faculty of Medicine, RWTH Aachen University, Pauwelsstr. 30, 52074 Aachen, Germany

**Keywords:** Calreticulin, Frameshift mutants, del52, Myeloproliferative neoplasms, MPN, Megakaryopoiesis, MPL, Degradation, Protein secretion, NF-E2

## Abstract

**Background:**

Somatic calreticulin (CALR), Janus kinase 2 (JAK2), and thrombopoietin receptor (MPL) mutations essentially show mutual exclusion in myeloproliferative neoplasms (MPN), suggesting that they activate common oncogenic pathways. Recent data have shown that MPL function is essential for CALR mutant-driven MPN. However, the exact role and the mechanisms of action of CALR mutants have not been fully elucidated.

**Methods:**

The murine myeloid cell line 32D and human HL60 cells overexpressing the most frequent CALR type 1 and type 2 frameshift mutants were generated to analyze the first steps of cellular transformation, in the presence and absence of MPL expression. Furthermore, mutant CALR protein stability and secretion were examined using brefeldin A, MG132, spautin-1, and tunicamycin treatment.

**Results:**

The present study demonstrates that the expression of endogenous *Mpl*, CD41, and the key megakaryocytic transcription factor NF-E2 is stimulated by type 1 and type 2 CALR mutants, even in the absence of exogenous MPL. Mutant CALR expressing 32D cells spontaneously acquired cytokine independence, and this was associated with increased *Mpl mRNA expression*, CD41, and NF-E2 protein as well as constitutive activation of downstream signaling and response to JAK inhibitor treatment. Exogenous expression of MPL led to constitutive activation of STAT3 and 5, ERK1/2, and AKT, cytokine-independent growth, and reduction of apoptosis similar to the effects seen in the spontaneously outgrown cells. We observed low CALR-mutant protein amounts in cellular lysates of stably transduced cells, and this was due to accelerated protein degradation that occurred independently from the ubiquitin-proteasome system as well as autophagy. CALR-mutant degradation was attenuated by MPL expression. Interestingly, we found high levels of mutated CALR and loss of downstream signaling after blockage of the secretory pathway and protein glycosylation.

**Conclusions:**

These findings demonstrate the potency of CALR mutants to drive expression of megakaryocytic differentiation markers such as NF-E2 and CD41 as well as *Mpl*. Furthermore, CALR mutants undergo accelerated protein degradation that involves the secretory pathway and/or protein glycosylation.

**Electronic supplementary material:**

The online version of this article (doi:10.1186/s13045-016-0275-0) contains supplementary material, which is available to authorized users.

## Background

Using exome sequencing, calreticulin (CALR) frameshift mutations were discovered in myeloproliferative neoplasms (MPN) and shown to be restricted to essential thrombocythemia (ET) and primary myelofibrosis (PMF) [1, 2]. Both neoplasms involve striking abnormalities of megakaryocytes [3, 4]. CALR, Janus kinase 2 (JAK2) V617F, and thrombopoietin receptor (MPL) mutations were mutually exclusive, suggesting that they all activate the JAK2-STAT signaling pathway to transform hematopoietic stem cells. While only exceptional cases of CALR-mutant polycythemia vera (PV) have been described in cases that were negative for JAK2V617F [5], 15–24 % and 25–35 % of patients with ET and PMF, respectively, carry a CALR mutation [3]. CALR frameshift mutations are classified according to the length of the somatic deletion or insertion in exon 9 of the *CALR* gene. Until now, 36 types of CALR mutants have been observed in MPN [2]. All of these mutants lead to a 1-bp frameshift and loss of the KDEL sequence and the original *CALR* stop codon [2]. The most frequent variants, the type 1 (c.1092_1143del) and type 2 (c.1154_1155insTTGTC) mutations, representing either a 52-bp deletion (p.L367fs*46; del52) or a 5-bp insertion (p.K385fs*47; ins5), respectively, account for approximately 80 % of all CALR mutations [1, 2]. Type 1 and 2 CALR mutations have been shown to carry prognostic relevance [6], but this was not found by all groups [7].

CALR is a chaperone which is localized in the endoplasmic reticulum (ER) and exhibits an N-terminal ER-signal sequence, a N-, P-, and C-domain, and the ER retrieval sequence KDEL [8]. CALR function regulates protein folding and quality control processes [9]. Furthermore, CALR strongly affects calcium (Ca^2+^) homeostasis in the ER/cytoplasm and thus Ca^2+^-dependent signaling through its P-domain (low Ca^2+^ capacity; high Ca^2+^ affinity) and C-domain (high Ca^2+^ capacity; low Ca^2+^ affinity) [8]. The modified C-terminus in CALR frameshift mutants comprises several additional triplets that were formerly part of the 3′UTR in wild-type (WT) CALR. Importantly, a large proportion of negatively charged amino acids in the C-domain of WT CALR converts into positively charged amino acids, abolishing proper Ca^2+^-binding [10].

While the function of CALR mutants in ET and PMF has remained unclear, recently, Marty et al. and Chachoua et al. have highlighted the necessity of the thrombopoietin (TPO) receptor MPL and its N-glycosylation to be essential for cellular transformation [11, 12]. Marty et al. established a retroviral mouse model of del52 and ins5, closely reflecting an ET phenotype and, in the case of CALR del52, also the progression to myelofibrosis [12]. Furthermore, two research groups have shown physical interaction of CALR mutants and MPL and the necessity of the positive electrostatic charge of the novel C-terminus for this interaction [13, 14]. Araki et al. presented a model by which the P-domain in WT CALR blocks MPL interaction [13]. This inhibitory function of the P-domain is abolished by the novel C-terminus in mutant CALR, thus enabling the N-domain to interact with the extracellular domain of MPL and leading to its dimerization and activation.

In the present study, we investigated the impact of CALR mutants on megakaryocytic transcription factors implicated in endogenous *Mpl* and CD41 expression. Moreover, we assessed CALR-mutant protein stability and secretion. We further confirmed MPL-dependence of CALR mutant-driven cell transformation and protection from apoptosis, as well as activation of critical signaling proteins including STAT5, STAT3, AKT, and ERK1/2. Collectively, our findings extend our understanding of CALR frameshift mutants’ cellular characteristics involved in pathogenesis and suggest that CALR mutants support megakaryocytic differentiation by MPL-dependent and MPL-independent mechanisms.

## Methods

### Patient samples and cDNA

RNA from patients carrying WT CALR or the ins5 mutant was isolated from the peripheral blood of MPN patients after written informed consent and ethics committee approval (EK2127/12). Complementary DNA (cDNA) from a patient with CALR del52 mutant was provided by Prof. S. Schnittger and Prof. T. Haferlach (Munich). The patient gave written informed consent to research studies, and the study was approved by the local ethics committee (05117) and adhered to the tenets of the Declaration of Helsinki. The wild-type and mutant CALR cDNA fragments used for vector cloning were obtained from patients’ RNA by reverse transcription polymerase chain reaction (RT-PCR) with random primers.

### Reagents and antibodies

The proteasome inhibitor MG132, tunicamycin, and brefeldin A (BFA) were purchased from Sigma-Aldrich (St. Louis, MO, USA). Ruxolitinib (LC Labs, Woburn, MA, USA), spautin-1 (Selleckchem, Houston, TX, USA), and tunicamycin were dissolved in DMSO. BFA was dissolved in 100 % methanol. TransIT-LT1 (Mirus, Madison, WI, USA) was used to transfect HEK293T cells according to the manufacturer’s instructions. Antibodies used in our study included polyclonal rabbit anti-mouse/human phospho-STAT5 (Tyr694), polyclonal rabbit anti-mouse/human phospho-STAT3 (Tyr705), monoclonal rabbit anti-mouse/human phospho-AKT (Ser473) (193H12), polyclonal rabbit anti-mouse/human phospho-p44/42 MAPK (Erk1/2) (Thr202/Tyr204), polyclonal rabbit anti-mouse/human p44/42 MAPK (Erk1/2), monoclonal rabbit anti-mouse/human LC3B (3868s) and monoclonal rabbit anti-mouse/human STAT3 (D3Z2G), which were obtained from Cell Signaling/New England Biolabs (Frankfurt, Germany). The mouse monoclonal HA-probe antibody (sc-7392), polyclonal goat anti-mouse/human AKT1/2 (sc-1619), monoclonal mouse anti-mouse/human NF-E2 (sc-365083), monoclonal mouse anti-mouse/human GAPDH (sc-32233), and polyclonal rabbit anti-mouse/human DNMT3B antibody (sc-20704) were ordered from Santa Cruz Biotechnology (Santa Cruz, CA, USA). Monoclonal rabbit anti-mouse/human calreticulin antibody (EPR3924) from Merck Millipore (Darmstadt, Germany) was used for total calreticulin detection. The mouse monoclonal antibody CAL2 (Dianova, Hamburg, Germany) against the novel C-terminal of the CALR mutants was used for CALR mutant (CALR mut) detection. The mouse monoclonal anti-flag antibody M2 (F3165) was ordered from Sigma-Aldrich. The antibody against STAT5A/B was a kind gift from Richard Moriggl (Ludwig Boltzmann Institute for Cancer Research (LBI-CR), Vienna, Austria) and was originally generated by Eurogentec, Cologne, Germany. The CD41-APC-eFluor®780 (MWReg30) antibody and its isogenic control (eBRG1) were obtained from eBioscience (San Diego, CA, USA). Polyclonal goat anti-rabbit immunoglobulins/HRP (P0448), polyclonal goat anti-mouse immunoglobulins/HRP (P0447), and polyclonal rabbit anti-goat immunoglobulins/HRP (P0160) antibodies were purchased from DAKO (Hamburg, Germany).

### DNA constructs and vectors

The flag-tagged cDNA of WT CALR, del52, and ins5 were cloned into the pMSCV-IRES-GFP vector and pcDNA5/FRT/TO vector (Life Technologies – Thermo Fisher Scientific, Paisely, UK) by Gateway cloning system (Life Technologies – Thermo Fisher Scientific). In case of the WT CALR, the KDEL sequence was cloned behind the flag-tag sequence. The cDNA of human MPL in the pMSCV-neo vector was kindly provided by Rebekka Schneider-Kramann. The C-terminally yellow fluorescent protein (YFP)-tagged cDNA of WT CALR, del52, and ins5 were cloned into the pMSCV-IRES-puromycin vector, and the KDEL sequence was cloned following the YFP-tag only in the WT CALR expression vector.

### Cell culture and retroviral transduction

32D cells were cultured in RPMI 1640 medium (Life Technologies – Thermo Fisher Scientific) supplemented with 10 % fetal bovine serum (FBS), 25 U/ml penicillin/streptomycin (Life Technologies – Thermo Fisher Scientific) and 10 % Walter and Eliza Hall Institute (WEHI) supernatant as source of interleukin-3 (IL-3). HEK293T cells were cultured in Dulbecco’s Modified Eagle’s Medium (DMEM, Biochrom, Berlin, Germany) supplemented with 10 % FBS and 25 U/ml penicillin/streptomycin. The HL60e cell line stably expressing ecotropic receptor was a gift from Herbert Strobl (Vienna) generated by Bradley Fletcher (FL, USA). The retroviral transduction was performed as previously described [15]. Briefly, Plat-E-packaging cells were transfected with pMSCV-IRES-GFP or pMSCV-IRES-neo/puro vectors containing the genes of interest and supernatants were collected after 24 and 48 h. Stable 32D cell lines were generated by three rounds of retroviral spin onto RetroNectin-coated (Takara Bio Europe/Clontech, France) six-well plates followed by selection by means of flow cytometry for GFP or G418/puromycin (InvivoGen, CA, USA) treatments.

### Proliferation assay

32D cells were plated in triplicate at a density of 2 × 10^5^ cells/ml and were cultured in WEHI-free RPMI medium. Living cells were manually counted every 24 h in a standard hemocytometer, excluding dead cells by trypan blue staining.

### Immunophenotypic analysis

Staining of 32D CALR cells for the presence of membrane-localized CD41 was performed using a Gallios flow cytometer (Beckman Coulter, Krefeld, Germany). Data were evaluated using FlowJo data analysis software (OR, USA).

### Preparation of cell lysates, SDS-PAGE, and immunoblotting

Cell lysates were produced with RIPA buffer containing 50 mM Tris pH 7.4, 150 mM sodium chloride, 1 mM EDTA, 1 % Triton-X, 15 % glycerol, 0.5 % sodium deoxycholate, and protease/phosphatase inhibitors. Denaturing of protein lysates was done at 65 °C for 5 min and separated by SDS-PAGE and transferred to polyvinylidene difluoride (PVDF) membrane (GE Healthcare, Frankfurt, Germany). Western blotting was performed overnight in Towbin transfer buffer (3 g Tris, 14.4 g glycine, 5 % methanol per liter ddH_2_O) at 100 mA. Ten percent BSA in TBS-I buffer (20 mM Tris–HCl, pH 7.6, 137 mM NaCl, 0.05 % IGEPAL) was used for membrane blocking. The primary antibody (1:1000) was incubated overnight at 4 °C, and the secondary antibody conjugated to HRP (1:2000) for 45 min with three times washing in between. Proteins were detected via chemoluminescence (Fusion SL, PeqLab). ImageJ software was used for protein quantification analysis [16].

### Ubiquitination assay

HEK293T cells were transiently transfected with 3 μg pcDNA5 vector expressing CALR WT and CALR del52 together with or without 4 μg pcDNA3-ubi-HA vector using TransIT-LT1 reagent (Mirus, Madison, USA). After 24 h, the medium was discarded and replaced by fresh DMEM containing 10 μM MG132 for 20 h, and cell lysates were generated. For the immune precipitation (IP) assay, 40 μl Protein G Sepharose (GE Healthcare, Freiburg, Germany) was mixed with 2 μg anti-flag antibody for 4 h at 4 °C and subsequently incubated with 1 mg lysate in 1 ml RIPA buffer overnight, rocking at 4 °C with three times washing using cold PBS in between. The sepharose was then washed three times with RIPA buffer and resuspended in Laemmli/RIPA solution. After denaturation, bound proteins were separated and prepared for Western blotting.

### MTT assay

32D cells were plated in triplicate at a density of 3 × 10^4^ cells/well in a 96-well plate after washing twice with PBS. The JAK inhibitor ruxolitinib was added to the medium (max. vol. of 100 μl) at the concentration of 1 μM. Controls were treated with DMSO. Cells were cultured in WEHI-free RPMI medium. The measurement of cell viability was performed 48 h later using 10 μl 3-(4,5-dimethylthiazol-2-yl)-2,5-diphenyltetrazolium bromide (MTT) (5 mg/ml H_2_O) per well. The 96-well plate was incubated in the dark at room temperature for 4 h, and 100 μl isopropanol-HCl solution per well was added. Samples were analyzed with a microplate reader at a wavelength of 550 nm (Kayto, RT-2100C).

### RT-qPCR

RNA isolation was performed by TRIzol/chloroform extraction (Trizol, Life Technologies, Darmstadt, Germany) using 6 × 10^6^ cells. One microgram RNA was used for cDNA synthesis. Quantitative RT-PCR was performed using the 7500 Fast Real-time PCR System (Applied Biosystems by Life technologies, Paisley, UK) with the SYBR Select Master Mix for CFX (Applied Biosystems). The sequences of primers used for RT-qPCR were as follows: GCGTAACAAAGGCAGCAGAG (*CALR* for), CGTCGTCGTCCTTGTAGTC (*CALR*-flag rev), GTGAGTCCCCTAGCTTGCTG (mu c-*Mpl* for), TAGCAGGTGTGAACGACAGG (mu c-*Mpl* rev), CCAATACGGCCAAATCCG (mu *Gapdh* for), CCAATACGGCCAAATCC (mu *Gapdh* rev), ACAGGTGCCTGAAAGGTTGC (mu *Nfe2* for), and ACCCTGCAGCTCAGTAATGG (mu *Nfe2* rev). The mRNA expression level of the target gene is determined in percentage of *Gapdh* using 2^-ΔCT.

### Concentration of 32D supernatant

32D MPL transduced cells expressing CALR WT or del52 were starved in FBS and WEHI-free RPMI medium at the density of 2 × 10^6^ cells/ml after washing twice with PBS. After starvation for 6 h, the cell viability was analyzed by cell analyzer CASY TTC (OLS, OMNI Life Science, Bremen, Germany) and the supernatant was collected and filtered (0.45 μM filter) to the concentrator Vivaspin 20 (Sartorius Stedim Biotech, Goettingen, Germany) followed by spinning at the speed of 4000×*g* at 4 °C for 30 min. Around 3.5 or 12 μg protein of the supernatant was used for the analysis of paracrine signaling, respectively.

### Statistical analysis

Statistical analysis was performed with the GraphPadPrism software using the two-tailed Student’s *t* test. Significant differences were determined by *P < .05, **P < .01, and ***P < .001; mean and standard deviation (SD) are indicated. The results are representative of at least three independent experiments.See Additional file 1 for supplementary material & methods.

## Results

### JAK-dependent spontaneous transformation of 32D del52 CALR cells

**Fig. 1:**
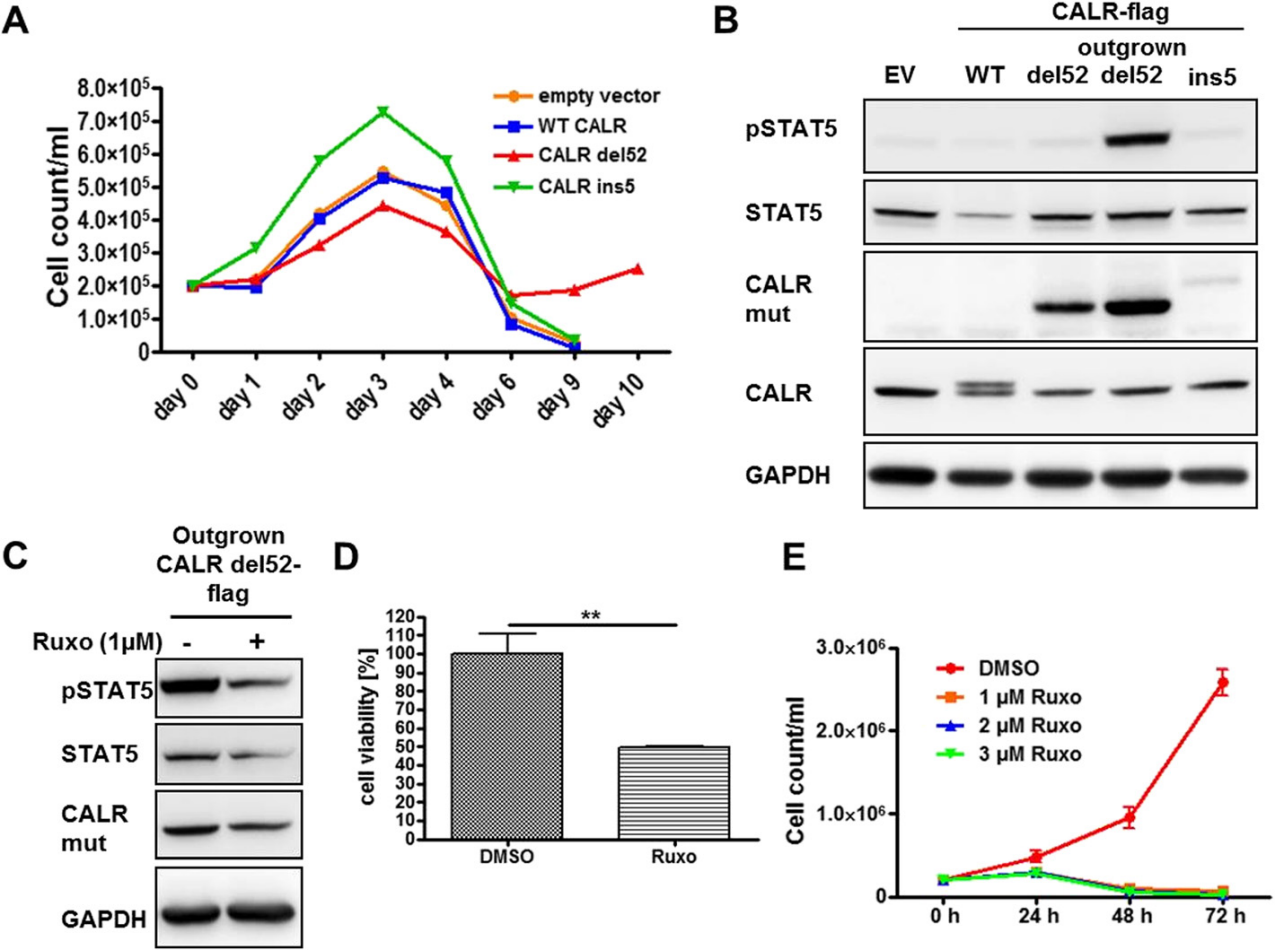
Spontaneously outgrown 32D CALR del52 cells are ruxolitinib (Ruxo) sensitive. **a** We performed growth assays with stably transduced 32D cells expressing empty vector (EV), WT CALR, del52 or the ins5 mutants (2 × 10^5^ cells/ml). Outgrowth of a CALR del52 expressing clone has been observed after 6 to 10 days. **b** 32D EV, CALR WT, CALR del52, CALR ins5, and outgrown del52 cells were starved for 16 h; lysates were prepared and subjected to SDS-PAGE and Western blotting. Indicated antibodies have been used for immunostaining. **c** Outgrown 32D CALR del52 cells were treated overnight with 1 μM Ruxo, and reduction of STAT5 phosphorylation could be detected in Western blotting. **d** In a MTT assay, outgrown 32D CALR del52 cells were treated with 1 μM Ruxo for 48 h, and cell viability was evaluated. The experiment was performed in triplicates. SD is indicated. **P < 0.01 **e** Outgrown 32D del52 cells were grown in WEHI-free medium (2 × 10^5^ cells/ml) supplemented with 1, 2, and 3 μM Ruxo or DMSO as control for up to 72 h. The cells were counted every 24 h. The experiment was performed in triplicates. SD is indicated

We analyzed the effect of stable ectopic expression of the two most prominent CALR mutants (type 1: del52, type 2: ins5) using IL3-dependent 32D and Ba/F3 cells, and analysis of these cells confirmed previous reports that this did not induce growth factor-independent proliferation in the majority of cells [14, 17]. However, after 5 days of starvation, a subpopulation of CALR del52 expressing 32D cells began to grow cytokine independently (Fig. 1a), and this was accompanied by increasing STAT5 phosphorylation (Fig. 1b). Moreover, these outgrown cells were sensitive to the JAK1/2 inhibitor ruxolitinib (Fig. 1c, d) which abrogated the growth of these cells completely (Fig. 1e). We did not observe outgrowth of a CALR ins5 mutant or CALR WT cell population.

Western blot analysis, using a CALR mutant-specific antibody [18], demonstrated the presence of the ectopically expressed CALR mutants, with the expression of ins5 being considerably lower than that of the del52 mutants (Fig. 1b). STAT5 phosphorylation was solely detected in the outgrown cell subpopulation (Fig. 1b).

### Upregulation of the megakaryocytic transcription factor NF-E2 in CALR-mutant cells

CALR mutations are restricted to MPN subtypes displaying aberrant megakaryopoiesis, such as ET, PMF, and post essential thrombocythemia-myelofibrosis (post-ET-MF) [3]. Megakaryopoiesis is regulated by megakaryocytic transcription factors as inducers of megakaryocytic differentiation and transcriptional activators of the *Mpl* promoter [19–22]. Therefore, we studied whether the expression of the megakaryocytic transcription factor NF-E2 was affected by the CALR mutants and whether it was increased in the outgrown 32D del52 cells.

**Fig. 2:**
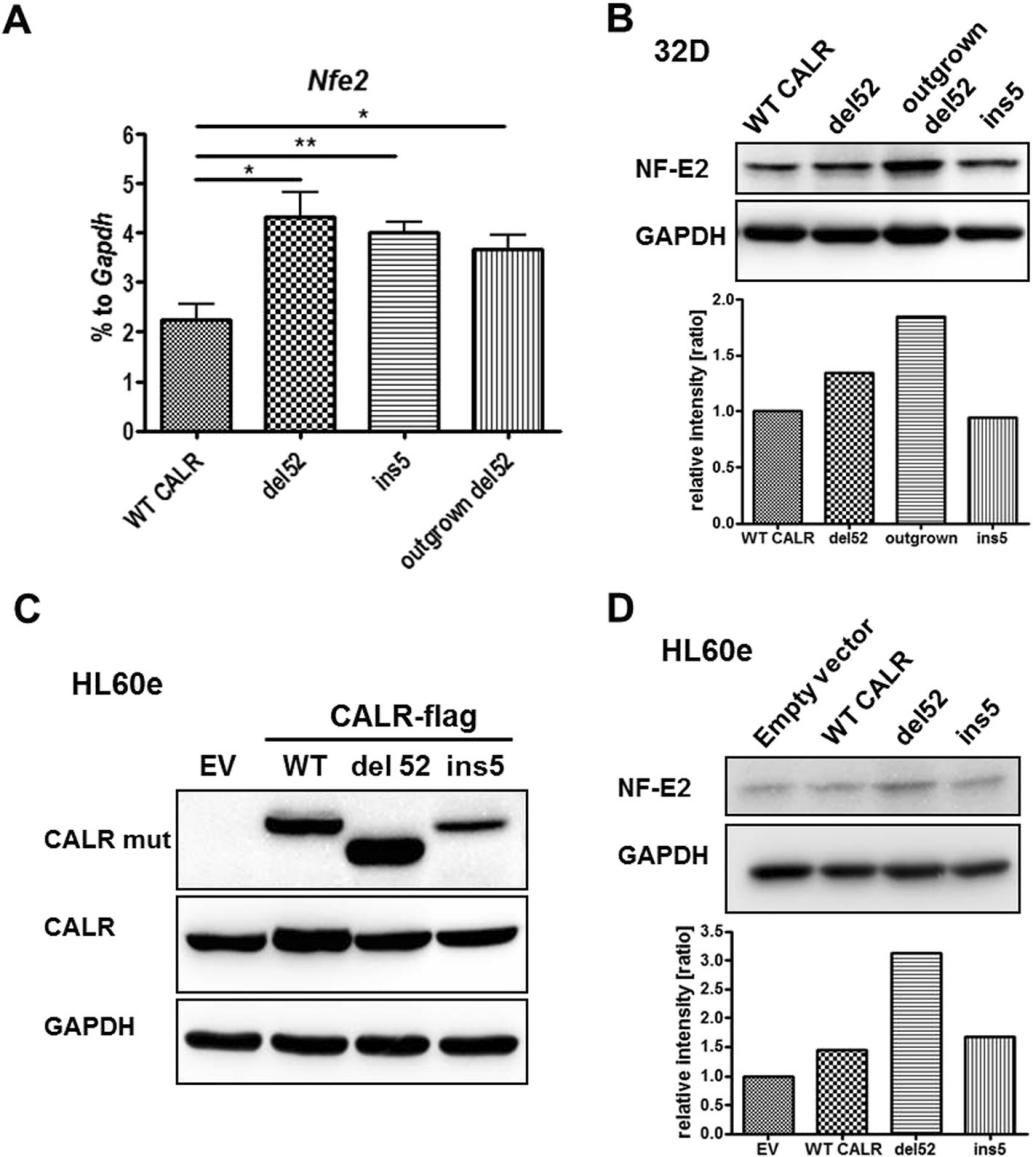
32D cells expressing CALR mutants show increased mRNA and protein levels of the megakaryocytic transcription factor NF-E2. **a** Detection of *Nfe2* mRNA expression by RT-qPCR in the indicated 32D cells. The experiments were performed in triplicates. SD is indicated. *P < 0.05, **P < 0.01, ***P < 0.001. **b** Lysates were prepared of 32D cells expressing WT CALR, del52, and ins5 mutant as well as of outgrown 32D del52 cells, and NF-E2 protein was detected in Western blotting. In **b** and **d**, GAPDH served as loading control and was used for the calculation of NF-E2 expression ratios. **c** HL60e EV (empty vector), CALR WT, CALR del52 and CALR ins5 cells were used to prepare lysates, and SDS-PAGE and Western blotting were performed. Indicated antibodies have been used for immunostaining. CALR mut antibody showed unspecific binding to ectopic WT CALR. **d** HL60e cells expressing empty vector (EV), WT CALR, del52, or ins5 were analyzed for NF-E2 expression, and expression ratios were calculated as in **b**

Interestingly, we detected a significant increase of *Nfe2* mRNA expression in the del52- and ins5-mutant expressing cells as well as the outgrown 32D del52 cells, when compared to 32D cells ectopically expressing WT CALR (Fig. 2a). We confirmed an increase of NF-E2 at the protein level in CALR del52 outgrown cells and to a lesser extent in del52 expressing cells, while ins5 mutant expressing cells showed no elevated NF-E2 protein (Fig. 2b). We generated human HL-60e cells expressing empty vector, WT CALR, CALR del52, or ins5 mutant to evaluate the findings in 32D cells (Fig. 2c). In these cells, WT CALR was detected not only by the conventional CALR antibody but also by the mutant-specific antibody, which was potentially due to drastic protein overexpression. The del52-induced increase of NF-E2 protein was confirmed in the stably transduced HL60e cells (Fig. 2d). Other transcription factors involved in megakaryopoiesis, such as *Gata1*, *Fli-1*, and *Ets-1* [20, 23], were not consistently altered, although Gata1 mRNA was significantly increased in del52-, ins5-, and outgrown del52-mutant 32D cells (Additional file 2: Figure S2a, b, c). We conclude that forced expression of CALR mutants alone is sufficient to induce upregulation of the megakaryocytic transcription factor NF-E2.

### CALR mutants trigger the expression of *Mpl* and CD41 in 32D cells

**Fig. 3:**
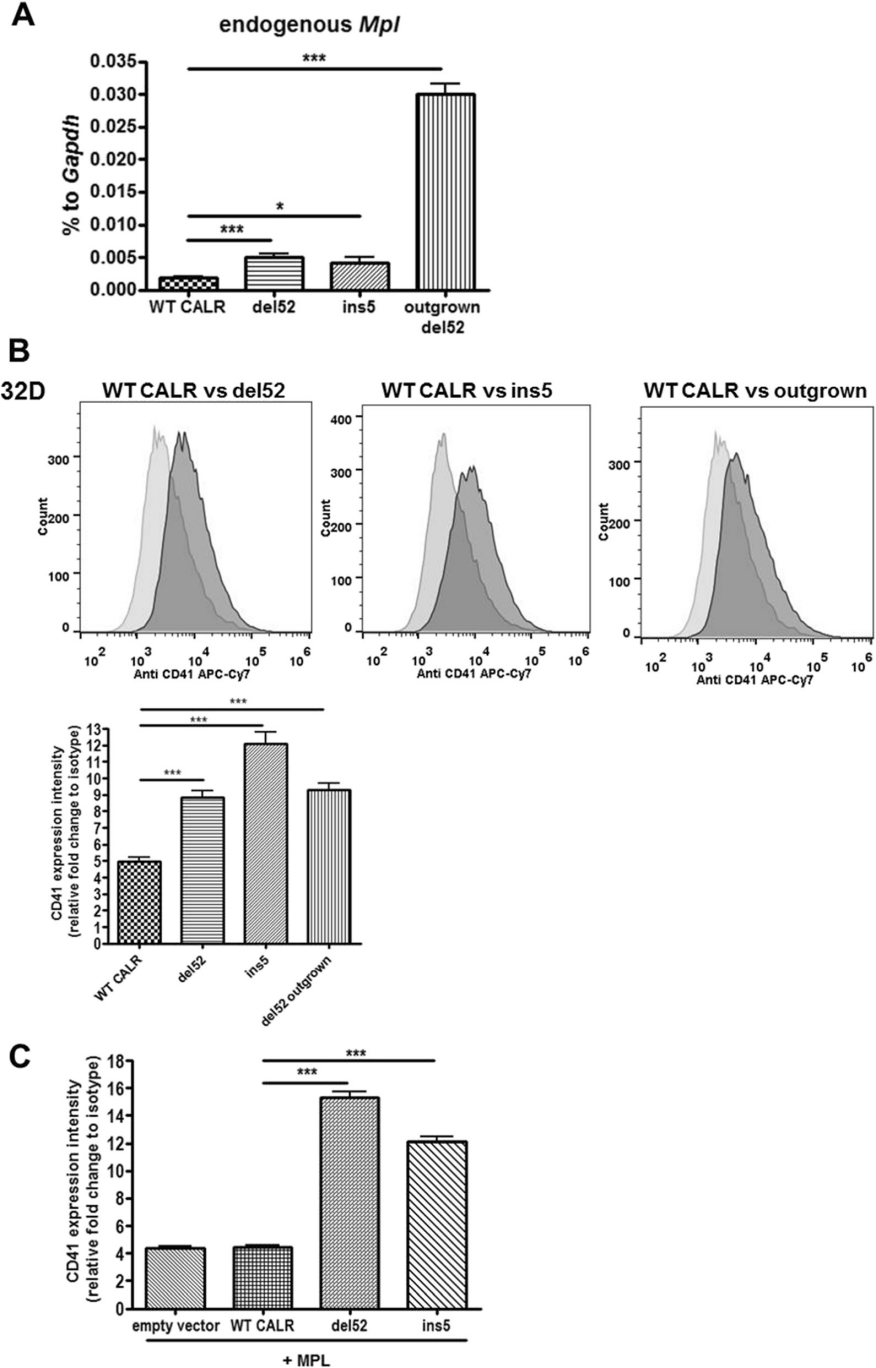
Endogenous *Mpl* as well as CD41 was upregulated by CALR-mutant expression. **a** RT-qPCR was used to detect *Mpl* mRNA amounts after RNA isolation of the indicated 32D cells followed by cDNA synthesis. Expression is depicted in percentage to *Gapdh*. Measurements were done in triplicates. SD is indicated. *P < 0.05 **b** FACS analysis of membrane-localized CD41 in the indicated 32D cell lines. Relative expression intensity was calculated using the mean fluorescent intensity (MFI) of triplicates. SD is indicated. ***P < 0.001 **c** As in **b**, the amount of CD41 was evaluated in 32D MPL cells expressing empty vector, WT CALR, del52, or ins5 using FACS. The experiment was performed in triplicates and the SD is indicated. ***P < 0.001

Having shown that NF-E2 was upregulated by CALR mutant expression, especially in the 32D outgrown del52 cells, we hypothesized that MPL expression may be upregulated in the CALR-mutant 32D cell lines as well as in the outgrown cells. Therefore, we analyzed endogenous *Mpl* expression in the transduced 32D cells and observed a significant increase of *Mpl* mRNA in the 32D del52 and ins5 mutant cells and in the outgrown del52 mutant cells (Fig. 3a). Interestingly, when we analyzed the expression of two other type-I cytokine receptors in del52 vs outgrown del52 cells, the EPO receptor (EPOR) and the G-CSF receptor (G-CSFR), known to be necessary for JAK2 V617F transforming activity [24], we detected a significant decrease of *Epo receptor* mRNA in the outgrown 32D del52 cells (Additional file 2: Figure S2d), while no *Gcsf* receptor mRNA was detected. Furthermore, we observed a significantly higher expression of endogenous *Mpl*, when we overexpressed human MPL in the 32D CALR cell lines with an emphasis on del52 expressing cells (Additional file 2: Figure S2e).

Moreover, flow cytometry confirmed increased surface expression of the integrin CD41 (Fig. 3b), a differentiation marker for megakaryocytes and platelets and a target gene of GATA, MAFB, and NF-E2 transcription factors [25, 26]. Ectopic MPL expression also increased CD41 surface expression; however, this occurred specifically in del52 and ins5 CALR-mutant but not WT CALR expressing or control cells (Fig. 3c). Thus, we hypothesize that CALR frameshift mutants alone initiate the differentiation towards the megakaryocytic lineage, which is then promoted by enhanced MPL activity. However, CALR mutants alone usually appeared unable to induce enough Mpl to transform cells to growth factor independence.

### CALR mutants show low protein stability, proteasome-independent degradation, and stabilization by MPL expression

**Fig. 4:**
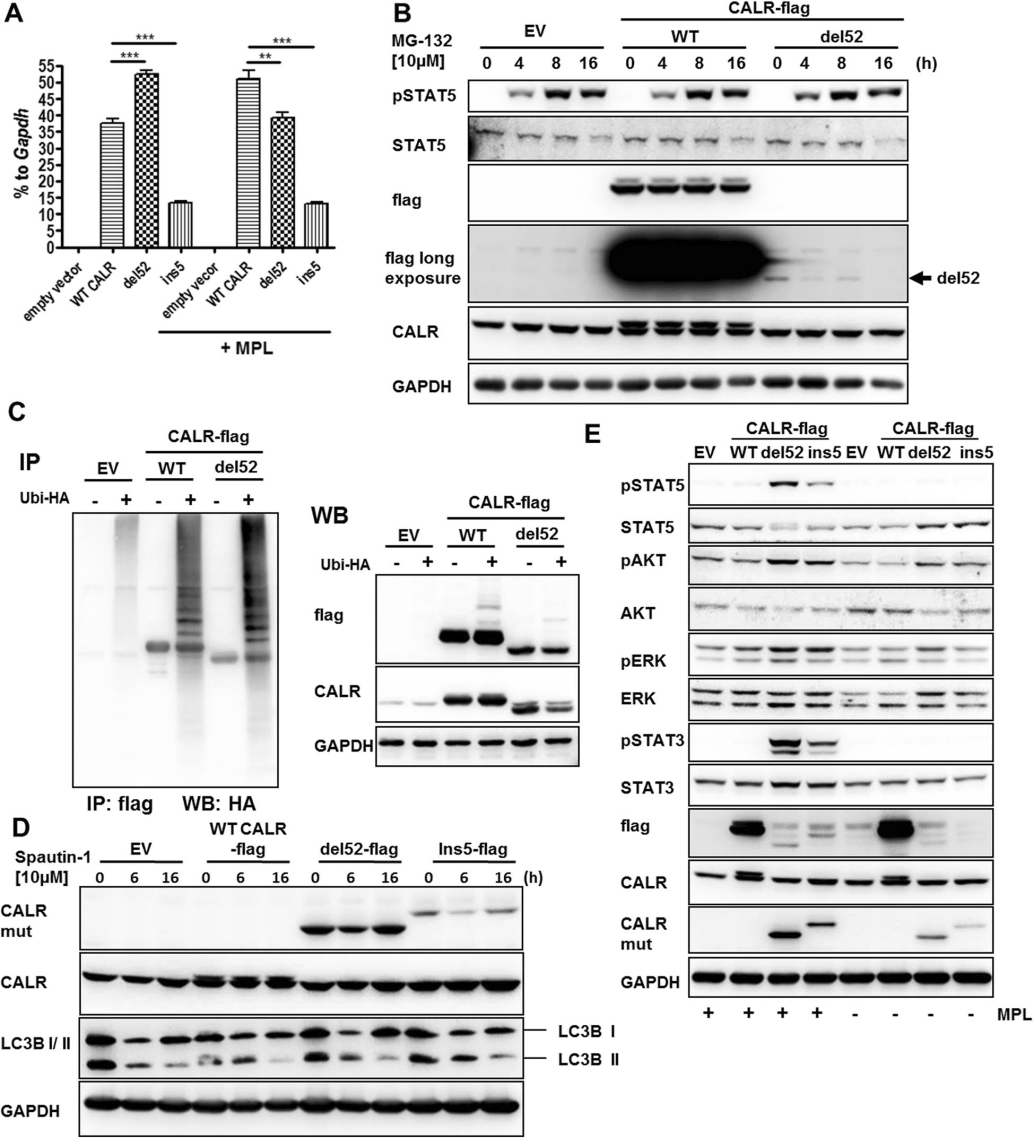
Degradation of instable CALR mutants is proteasome-independent and mutants get stabilized by MPL expression. **a** The expression of indicated *CALR-flag* constructs in 32D cells +/− ectopic MPL was confirmed by RT-qPCR. The expression is depicted as percentage to *Gapdh*. Experiments were performed in triplicates. Mean and SD are indicated. **P < 0.01, ***P < 0.001. **b** 32D empty vector (EV), WT CALR-positive, or del52-positive cells were treated with 10 μM MG132 for indicated periods of time. Afterwards, cells were starved in WEHI-free medium for 4 h and lysates were prepared for SDS-PAGE and Western blotting. Indicated antibodies were used for immunodetection to show protein stability. **c** HEK293T cells were transfected with HA-tagged ubiquitin and the indicated CALR-flag constructs. After 24 h, 10 μM MG132 was added for 20 h and protein lysates were prepared. Immunoprecipitation (IP) was performed with flag antibody followed by SDS-PAGE and Western blotting. In addition, whole cell lysates were used for Western blotting. **d** Autophagosomal inhibition was performed in 32D EV, WT CALR, del52, and ins5 expressing cells. The 32D cell lines were treated with the inhibitor spautin-1 for the indicated time. Lysates were prepared, and SDS-PAGE and Western blotting were performed. Antibodies detecting mutated CALR (CALR mut), CALR, LC3I-II, and GAPDH were used for immunostaining. LC3I-II served as control for successful autophagosomal inhibition. **e** 32D cells expressing EV, WT CALR, del52, or ins5 mutant +/− MPL receptor were starved overnight. Lysates were prepared and subjected to SDS-PAGE and Western blotting. The PVDF membrane was subjected to the indicated antibodies

We observed significantly lower protein amounts of CALR del52 and ins5 mutants than WT CALR protein in cellular lysates of transduced cells (Fig. 1b) although CALR mRNA was highly expressed (Fig. 4a). This was observed when Western blotting was performed using an antibody recognizing either the CALR C-terminus or the FLAG-tag but not when using a CALR mutant-specific antibody, suggesting that the mutant CALR proteins had undergone a conformational change. CALR ins5 mRNA and protein expression were consistently lower than that of del52 (Fig. 1b and Fig. 4a). We generated a C-terminal truncation mutant (ΔC, truncation of the entire novel C-terminal part in del52 mutant) and confirmed CALR antibody binding, showing that the specific epitope of the antibody is present in the CALR del52 and ins5 mutants (Additional file 3: Figure S3a). To evaluate the possibility of rapid proteasomal degradation of mutant CALR, we treated 32D cells with MG132, a proteasomal inhibitor (Fig. 4b). However, this did not lead to an increase of CALR del52 protein but rather a decrease, while WT CALR protein levels remained stable. We used pSTAT5 staining as a positive control for successful proteasomal inhibition by MG132, as published [27, 28]. Furthermore, ubiquitination of WT CALR and the del52 mutant was not different (Fig. 4c). Next, we analyzed if autophagosomal/lysosomal degradation was the reason for low CALR mutant abundance. We treated the 32D CALR cell lines with the autophagy inhibitor spautin-1 to block the fusion of autophagosomes with lysosomes. The block of the conversion from LC3B I to II served as control for successful autophagy inhibition. No increase of mutant CALR protein was observed upon inhibition of autophagy (Fig. 4d). To exclude the possibility that the lack of CALR mutant protein detection was due to an artifact in denaturing SDS gels, native PAGE gels were used, showing that detection of mutant CALR was not improved in native PAGE analysis (Additional file 3: Figure S3b).

Simultaneous ectopic expression of human MPL induced cytokine-independent proliferation and suppressed apoptosis in 32D cells ectopically expressing CALR del52 or the CALR ins5 mutant but not empty vector (EV)- and CALR WT-transduced 32D cells (Additional file 4: Figure S4a, b), as has been described previously [11–14]. No cytokine-independent proliferation and activated downstream signaling was observed by simultaneous ectopic expression of human EPOR (Additional file 4: Figure S4c, d). Starved 32D MPL cells expressing CALR mutants but not CALR WT showed constitutive phosphorylation of STAT5, STAT3, AKT, and ERK1/2 (Fig. 4e), and cells did not respond to recombinant TPO unless they overexpressed MPL (Additional file 4: Figure S4e). TPO-induced ERK1/2 phosphorylation was slightly increased in 32D del52 mutant cells in the absence of ectopic MPL expression, consistent with increased endogenous MPL expression, as suggested by the observed elevated mRNA levels (Fig. 3a). MPL protein was not evaluable due to lack of suitable antibodies. Protein levels of del52 and ins5 CALR were hardly detectable, using the flag and the overall CALR antibody, while WT CALR was readily detected (Fig. 4e). Importantly, MPL expression led to an increase of del52 and ins5 protein (Fig. 4e; CALR mut). A similar effect was observed in the outgrown 32D del52 CALR cells (Fig. 1b), again confirming our data of *Mpl* upregulation in the outgrown cells (Fig. 3a). Furthermore, the expression of del52 and ins5 mutants in 32D cells without exogenous MPL led to slightly increased AKT phosphorylation (Fig. 4e).

We conclude that CALR mutants are less detectable than their WT counterparts. This may either be due to decreased protein stability of CALR mutants or changes in protein structure that may prevent efficient binding of CALR antibody or the flag-specific antibody. MPL significantly enhanced protein detection of CALR mutants (Fig. 4e).

### Block of membrane trafficking and protein glycosylation inhibits CALR mutant-induced MPL signaling and increases cytoplasmic CALR mutant protein

**Fig. 5:**
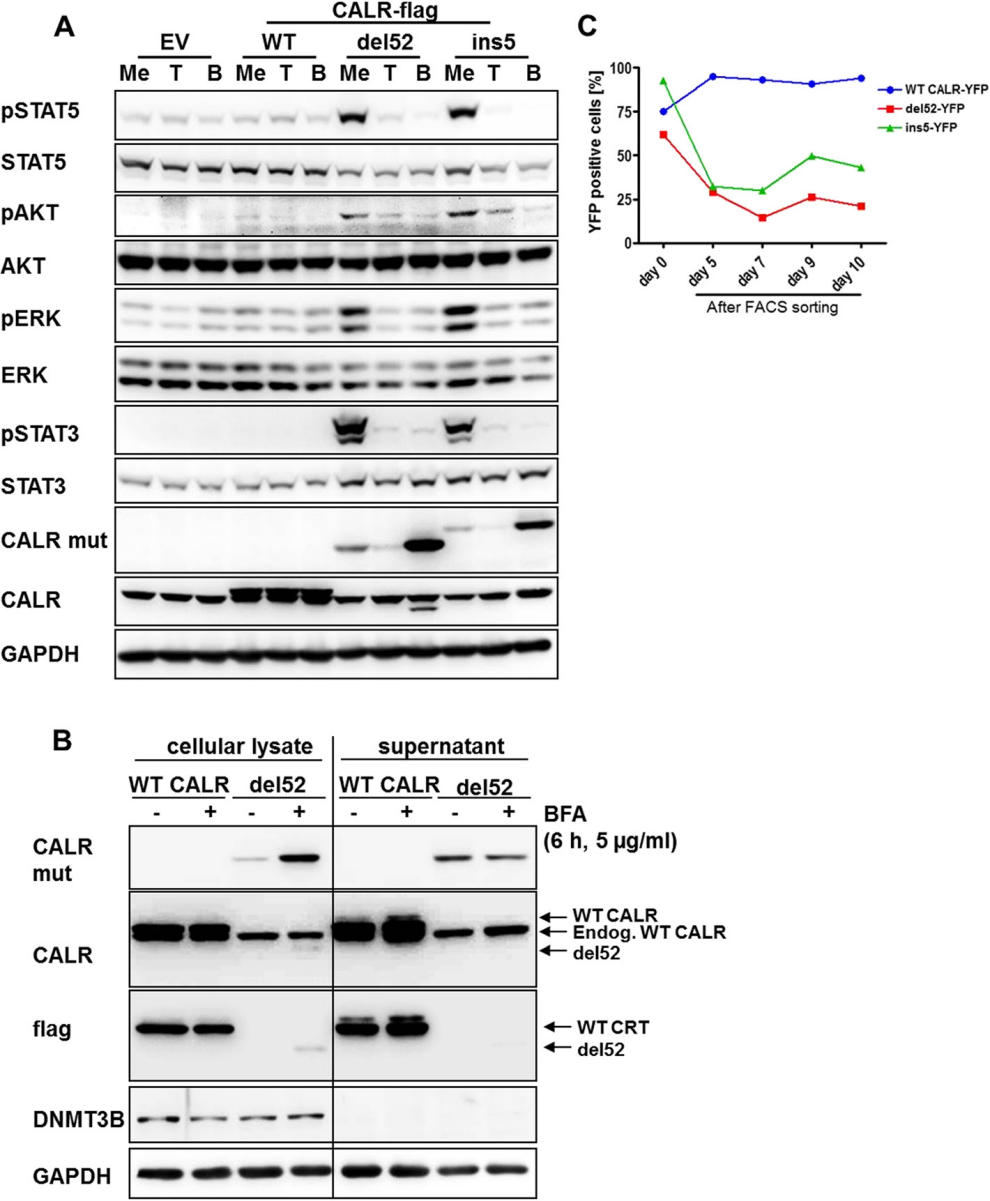
Glycosylation of MPL is essential for the activatory mechanism of CALR mutants, which are strongly secreted. **a** Empty vector (EV), WT CALR, del52, and ins5 expressing 32D MPL cells were treated with tunicamycin (*T*; 10 μg/ml), brefeldin A (*B*; 5 μg/ml), or the solvent methanol (*Me*) as control. Lysate preparation, SDS-PAGE, and Western blotting were performed. Indicated antibodies were used to confirm downstream signaling and CALR expression. GAPDH staining served as loading control. **b** Concentrated supernatants (30 μg protein) of indicated 32D cells, which were treated for 6 h with 5 μg/ml BFA or left untreated, were applied to SDS-PAGE followed by Western blotting and compared to the appropriate cellular lysates (30 μg protein). **c** 32D MPL cells expressing C-terminally YFP-tagged WT CALR, del52, or ins5 were FACS sorted for equal YFP-intensities and YFP signal was monitored until 10 days after sorting. Strong decrease of CALR del52-flag-YFP and ins5-flag-YFP could be observed

We and others have shown that the constitutively active receptor tyrosine kinase (RTK) FLT3-ITD activates STAT5 in the ER independently from its status of glycosylation [29, 30]. We asked whether MPL glycosylation and intracellular trafficking to the plasma membrane is necessary for CALR mutant-mediated MPL/JAK activation. Therefore, we treated 32D MPL cells expressing EV, CALR WT, del52, or ins5 with BFA. BFA has been shown to cause redirection from Golgi to the ER and disassembly of the Golgi apparatus as well as blocking of complex asparagine (N-) glycosylation [31]. In addition, tunicamycin was used to completely abolish N-glycosylation of proteins. After 8 h of BFA (5 μg/ml) or tunicamycin (10 μg/ml) treatment, phosphorylation of STAT5, STAT3, AKT, and ERK1/2 was strongly reduced, highlighting the importance of MPL glycosylation for CALR mutant-triggered downstream signaling (Fig. 5a).

Interestingly, BFA but not tunicamycin treatment significantly enhanced the amount of del52 and ins5 mutant protein in the whole cell lysates, as compared to vehicle treatment (Fig. 5a; CALR mut). We considered an increased secretion of CALR del52 and ins5 to be the reason for the low protein amount of CALR mutants in cellular lysates. Therefore, we collected the supernatant and the cell pellets of WT CALR or del52 expressing 32D MPL cells after addition of BFA or methanol control for 6 h. Again, we detected an increase of del52 mutant in the cellular lysates after BFA vs. vehicle treatment (Fig. 5b). Furthermore, we observed higher amounts of del52 in the supernatant in comparison to lysates, confirming a high secretion rate of CALR del52 (Fig. 5b; CALR mut, -BFA). WT CALR showed an equal extra- and intracellular pattern of distribution. DNMT3B (DNA (cytosine-5-)-methyltransferase 3 beta) staining confirmed the absence of contamination of the supernatant by cellular proteins.

To differentiate whether low CALR protein detection in CALR-mutant cells was due to low stability of the protein or due to low affinity of the CALR antibody, we fused WT CALR, del52, and ins5 proteins C-terminally to YFP and generated stably expressing 32D MPL cells. After sorting for high YFP levels in 32D MPL cells, del52 CALR-YFP and ins5 CALR-YFP cells were outcompeted by their normal WT CALR-YFP counterparts (Fig. 5c). At the same time, YFP-tagged CALR del52 in 32D MPL cells still activated STAT5 and conferred factor-independent cell growth (Additional file 5: Figure S5a, b). These results show that CALR del52 and ins5 proteins exhibit decreased stability, most likely together with increased secretion of del52-YFP and ins5-YFP protein.

**Fig. 6:**
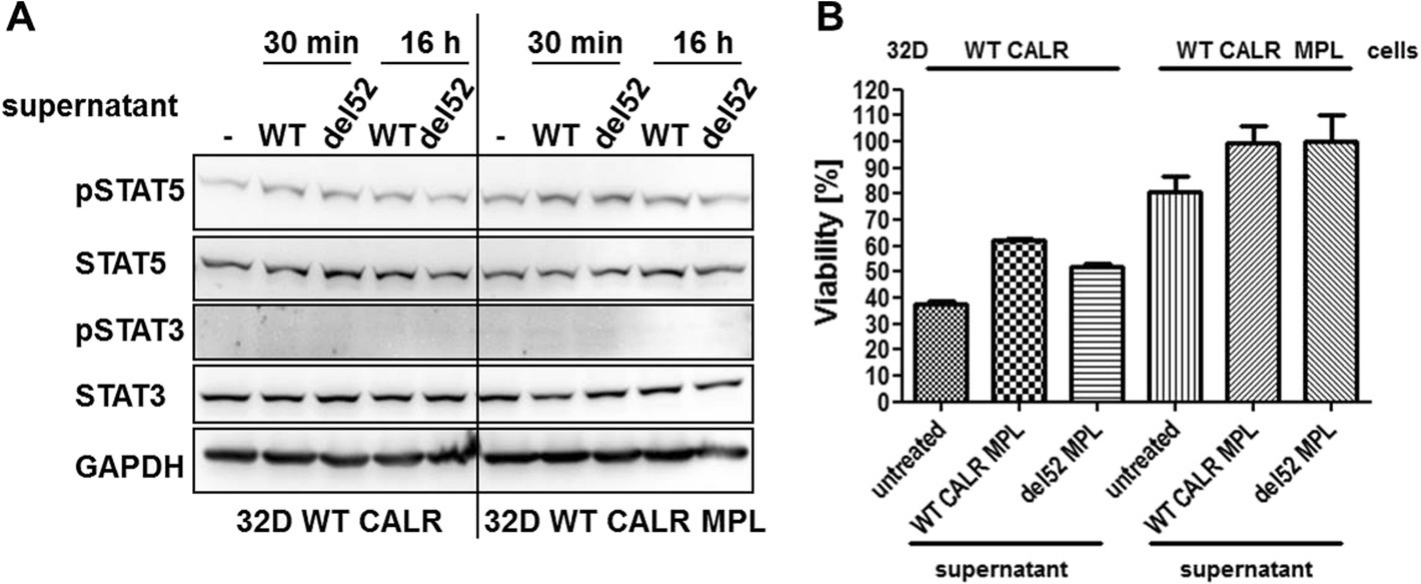
Secreted CALR del52 protein shows no paracrine function in 32D MPL cells. **a** 32D WT CALR +/− MPL cells were cultured with the indicated supernatants (12 μg of protein) for 30 min or 16 h before preparation of lysates to analyze STAT5 and STAT3 phosphorylation in Western blotting. **b** MTT assays were performed to analyze viability of 32D WT CALR +/− MPL cells cultured in FBS and WEHI-free medium for 48 h treated with the indicated supernatants (3.5 μg/100 μl). Relative viability in percent was calculated by determination of the values gained by the 32D MPL WT CALR cells + del52 supernatant approach as 100 %

WT CALR has been published to be secreted into the extracellular matrix and to have immune-modulatory functions [9, 32]. Because of our observation of highly secreted CALR mutant protein and its ability to interact with MPL, which had been also documented by Araki et al. and Marty et al. [12, 13], we analyzed possible paracrine signaling of the CALR del52 mutant protein. To do this, we added concentrated supernatant of 32D MPL CALR del52 cells onto 32D −/+ MPL cells expressing WT CALR. After 30 min and 16 h, we did not detect any activation of STAT5 or STAT3 in the exposed cells (Fig. 6a). In addition, 32D MPL del52 cell supernatant had no influence on the viability of 32D MPL WT CALR cells in IL3-free medium using an MTT assay (Fig. 6b).

## Discussion

In this study, we demonstrate the capability of the most frequent CALR type 1 and type 2 frameshift mutants to induce an increase of endogenous *Mpl*, *CD41*, and *Nfe2* expression in 32D cells, suggesting that these mutants may prime hematopoietic stem cells for megakaryocytic differentiation. Endogenous MPL, CD41, and NF-E2 mRNA and protein expression was increased by CALR del52 even in the absence of ectopic human MPL expression as well as in spontaneously outgrown 32D del52 CALR cells (Figs. 2 and 3; Additional file 2: Figure S2d). Although we cannot fully exclude a CALR del52-independent effect in the outgrown cells, the fact that spontaneous outgrowth occurred in our own experiments as well as in those published by Klampfl et al. [2] suggests that CALR del52 was responsible for the outgrowth of 32D cells. The increase of *Mpl* expression and stabilization of CALR del52 protein in the outgrown cells suggests that cytokine independence and ruxolitinib sensitivity were induced by similar mechanisms as in the CALR mutant-transduced 32D cells ectopically overexpressing MPL. Using Sanger sequencing, we excluded activating mutation in the *Jak2* (e.g., *V617F*) or *c-mpl* (e.g., *W506L*) *gene* in the outgrown 32D cells. Nevertheless, we cannot exclude the possibility that cells with spontaneously higher MPL or NF-E2 levels were selected in the presence of the CALR mutants, as opposed to direct *Mpl* and *Nfe2* induction by the CALR mutants.

There were differences between del52- and ins5-mutant expressing cells: MPL expression was inconsistent in CALR ins5 mutant expressing 32D cells (Fig. 3a and Additional file 2: Figure S2e), and GATA-1 mRNA levels did not correlate with protein except for 32D CALR ins5 cells (Additional file 2: Figure S2 c). In general, the effect of ins5 was less prominent than that of del52 except for CD41 upregulation. No outgrowth of 32D CALR ins5 cells was observed, potentially due to low protein expression. However, the weaker effect of ins5 mutants may also be due to negatively charged amino acids in the C-terminus which are missing in del52. Together, these differences may also at least in part explain clinical data that have associated ins5 mutants with a less severe phenotype than CALR del52 [12].

Our data suggest that CALR del52 and ins5 increase megakaryocytic differentiation by the alteration of the transcriptional program, although we can only illustrate the initializing components of megakaryocytic differentiation in our model system. These data are in line with observations from Marty et al. in a retroviral mouse model of CALR mutants resulting in an ET and post-ET-MF phenotype [12] and are supported by a recent study of Nivarthi et al. [33]. It is known that megakaryocytes arise from bipotent megakaryocytic-erythroid progenitors (MEP) [20]. Therefore, the reduction of *EpoR* mRNA together with the increase of *Mpl* that we observed in the outgrown 32D del52 cells suggests a differentiation shift from the erythrocytic to the megakaryocytic lineage.

How the CALR mutants del52 and ins5 led to the upregulation of megakaryocytic markers in 32D cells is not clear, but we detected an increase of AKT phosphorylation in del52 and ins5 expressing 32D cells in the absence of ectopic MPL expression (Fig. 4e). It was reported that active AKT positively influences megakaryopoiesis [34, 35]. In addition, the knockout of the negative regulator of PI3K/AKT activity PTEN results in a significant increase of platelets [36]. Therefore, active AKT could be an important mediator of CALR mutant-induced effects in our cell system and in hematopoietic stem cells/megakaryocytes.

CALR del52 and ins5 proteins showed low abundance, and we were able to exclude ubiquitin-triggered proteasomal degradation (Fig. 4b, c). In addition, our data suggests that autophagy or lysosome-dependent degradation [37] were not involved (Fig. 4d). Furthermore, decreased protein stability was substantiated by our novel YFP fusion CALR mutants (Fig. 5c). Experiments from Chachoua et al. using cycloheximide had already suggested decreased protein stability [11], which seems to be due to the presence of the new C-terminal domain [17]. We demonstrated that degradation was partially antagonized by overexpression of MPL, suggesting a stabilizing effect of MPL on CALR mutants, together with reduced CALR mutant protein secretion due to strong MPL interaction, described here and by Araki et al. [13]. This resulted in enhanced CALR mutant protein detection in cellular lysates (Fig. 4e; Additional file 4: Figure S4e). Similar stabilization has been observed in the outgrown 32D del52 cells (Fig. 1b).

Chachoua et al. had mutated all four N-glycosylation sites in the extracellular domain of MPL and shown strong reduction of STAT5 transcriptional activity in the presence of CALR mutants [11]. Therefore, it is tempting to speculate that binding of CALR mutants to the glycosylated TPO receptor in the lumen of the ER, Golgi, and transport vesicles may trigger receptor dimerization and JAK activation. Importantly, and in line with this hypothesis, we have observed a significant increase of CALR mutant protein upon BFA treatment of 32D MPL cells, while no increase of WT CALR was detected. BFA blocks the anterograde transport from the ER to the Golgi apparatus, leading to the collapse of Golgi stacks and accumulation of proteins in the ER [38, 39]. Consequently, it prevents protein secretion [40]. Tunicamycin treatment had no effect on del52 detection, maybe due to tunicamycin-induced unfolded protein response triggering ER-associated protein degradation (ERAD) [41].

We detected higher amounts of secreted CALR del52 mutant in the supernatant in comparison to whole cell lysates, which was blocked by BFA treatment (Fig. 5b). It is commonly accepted, albeit still a matter of debate, that WT CALR mediates cellular function from the cell surface and extracellular matrix, although the exit channels from the ER are still unknown [9]. Importantly, ER depletion of Ca^2+^ led to increased secretion and membrane localization of WT CALR [42, 43]. The occurrence of mutant CALR alters calcium homeostasis [10, 32], which could be one of the reasons for the observed increase of CALR mutant secretion. In addition, the KDEL sequence of WT CALR needs to be masked, negligible in CALR mutants. Although CALR mutants lack the ER retention signal KDEL, their localization is still primarily in the ER and Golgi apparatus, due to the ER-signal sequence in the N-terminus, where the MPL presumably gets activated [1, 11]. Nevertheless, activation of glycosylated membrane-standing MPL by CALR mutants cannot be excluded.

A paracrine function of CALR mutants in neighboring cells was hypothesized, as discussed already for WT CALR [9]. Nevertheless, in our experiments as well as in the work of Araki et al., no paracrine signaling of mutant CALR was detected [13]. As this absence of paracrine signaling could be due to low CALR mutant concentration in the supernatant, generation of recombinant CALR-mutant protein would be of interest. In addition, in vivo secretion of CALR mutants into the extracellular matrix of bone marrow and spleen would provide further ways of action. Thus, intracellular trafficking as well as enhanced secretion may be the major reasons for the low protein availability of mutant CALR, most likely in combination with proteasome-independent CALR mutant degradation.

## Conclusions

Our study shows that CALR mutants upregulate the key megakaryocytic factor NF-E2, the megakaryocytic surface marker CD41, and the thrombopoietin receptor MPL. These findings are important in light of the fact that CALR mutants occur predominantly in essential thrombocythemia and primary myelofibrosis, two malignancies that are characterized by aberrant megakaryopoiesis. We determine proteasome- and autophagosome/lysosome-independent degradation and enhanced secretion as the explanation for low cellular protein abundance and state MPL-triggered CALR-mutant stabilization. These findings significantly increase our understanding of CALR-mutant characteristics and highlight potential mechanisms of ET/MF pathogenesis.

## Ethics approval and consent to participate

RNA from patients was isolated from the peripheral blood of MPN patients after written informed consent and ethics committee approval (EK2127/12). cDNA from a patient with CALR del52 mutant was provided by Prof. S. Schnittger and Prof. T. Haferlach (Munich). The patient gave written informed consent to research studies, and the study was approved by the local ethics committee (05117) and adhered to the tenets of the Declaration of Helsinki.

## Consent for publication

Not applicable.

## Additional files

Additional file 1: Figure S1. Supplementary material & methods.

Additional file 2: Figure S2. Regulation of megakaryocytic factors by CALR mutants in murine and human cell lines. a Detection of *Fli-1* and *Ets-1* or b *Gata1* mRNA expression by RT-qPCR in the indicated 32D and HL60e cells. The experiments were performed in triplicates. Mean and SD are indicated. ***P < 0.001. c 32D expressing WT CALR, CALR del52, CALR ins5 cells, and outgrown 32D del52 cells were used to prepare lysates. SDS-PAGE and Western blotting were performed. An antibody for the detection of GATA-1 protein was used for immunostaining. GAPDH served as loading control and was used for the calculation of GATA-1 expression ratios. d RT-qPCR was used to detect *Mpl*, *EpoR*, and *G-csfR* mRNA amounts after RNA isolation of the indicated 32D cells followed by cDNA synthesis. Expression is depicted in percentage to *Gapdh*. Measurements were done in triplicates. Mean and SD are indicated. *P < 0.05. e Analysis of endogenous *Mpl* expression by RT-qPCR to compare 32D EV, WT CALR, CALR del52, and CALR ins5 expressing cells +/− ectopic MPL expression. Experiments were performed in triplicates. Mean and SD are indicated. **P < 0.01, ***P < 0.001. (PDF 55 kb)

Additional file 3: Figure S3. Missing CALR-mutant detection in Western blotting is not due to loss of the antibody epitope or an SDS-PAGE artifact. a HEK293T cells were transiently transfected with expression vectors for flag-tagged WT CALR, CALR del52, a C-terminal truncation mutant (ΔC; aa R366) and empty vector. Twenty-four hours after, transfection lysates were generated and SDS-PAGE followed by Western blotting were performed. The indicated antibodies were used for immunostaining. The used CALR antibody bound to the CALR ΔC mutant. b Native page followed by Western blotting was performed with non-denatured protein lysates of 32D CALR cells. The PVDF membrane was stained with CALR antibody, and the membrane was stripped and stained with a flag-specific antibody. (PDF 67 kb)

Additional file 4: Figure S4. Mutant CALR grants factor-independence, protects from apoptosis, and activates downstream signaling in an MPL-dependent manner. a A proliferation assay was performed with the indicated cell lines (2 × 10^5^ cells/ml). 32D MPL cells were counted every 24 h for 4 days. The cell counts are mean values of triplicates. b Empty vector, WT CALR, CALR del52, and ins5 expressing 32D cells were seeded in a density of 5 × 10^5^ cells/ml and grown for 48 h in WEHI-free medium. Apoptosis was analyzed by flow cytometry after staining with Annexin V-APC and 7-AAD. Mean and SD are indicated. *P < 0.05, **P < 0.01, ***P < 0.001. c The indicated 32D cell lines were stably transduced with the EPOR and a proliferation assay was performed (2 × 10^5^ cells/ml). The cells were counted every 24 h for 4 days. The cell counts are mean values of triplicates. d After 18 h starvation the indicated 32D cell lines were stimulated for 15 min with 1 U/ml EPO and lysates were prepared. SDS-PAGE, Western blotting and immunostaining with the indicated antibodies was performed. GAPDH served as loading control. e Stably transduced 32D cells with the indicated CALR-flag constructs +/− MPL were starved for 16 h and stimulated with 20 ng/ml human TPO for 15 min. Lysates were prepared and subjected to SDS-PAGE and immunoblotting using antibodies against phospho-STAT5, phospho-ERK1/2, phospho-STAT3, ERK1/2, STAT5, STAT3, and mutated CALR (CALR mut). GAPDH served as loading control. (PDF 172 kb)

Additional file 5: Figure S5. Expression of the C-terminally YFP-tagged CALR del52 mutant leads to STAT5 phosphorylation and cytokine-independent growth of 32D MPL cells. a 32D cells stably transduced with WT CALR-flag-YFP, CALR del52-flag-YFP, or empty vector (EV) −/+ MPL were WEHI-starved for 16 h and protein lysates were prepared. SDS-PAGE and Western blotting was performed and the indicated antibodies were used for immunodetection. YFP was detected using a GFP-specific antibody, and GAPDH served as loading control. b The indicated 32D MPL cells were cultured in WEHI-free medium (2 × 10^5^ cells/ml). The cells were counted every 24 h for 3 days. The cell counts are mean values of triplicates. (PDF 53 kb)

## Abbreviations

BFA: brefeldin A
bp: base pair
Ca^2+^: calcium
CALR: calreticulin
ER: endoplasmic reticulum
ERAD: ER-associated protein degradation
ET: essential thrombocythemia
FBS: fetal bovine serum
MPN: myeloproliferative neoplasm
MTT: 3-(4,5-dimethylthiazol-2-yl)-2,5-diphenyltetrazolium bromide
neo: neomycin
PMF: primary myelofibrosis
post-ET-MF: post essential thrombocythemia-myelofibrosis
puro: puromycin
PV: polycythemia vera
PVDF: polyvinylidene difluoride
RTK: receptor tyrosine kinase
RT-PCR: reverse transcription polymerase chain reaction
Ruxo: ruxolitinib
TPO: thrombopoietin
WT: wild type
YFP: yellow fluorescent protein
