# Supplementary material for: Calreticulin-mutant proteins induce megakaryocytic signaling to transform hematopoietic cells and undergo accelerated degradation and Golgi-mediated secretion
Source: J Hematol Oncol. 2016 May 13;9:45. doi: 10.1186/s13045-016-0275-0 (PMC4894373; doi:10.1186/s13045-016-0275-0)
Supplement: Supplementary file 1 — Supplementary material & methods. [file 13045_2016_275_MOESM1_ESM.pdf]

Additional File 1: Supplementary material and methods (.pdf)

DNA constructs and vectors

The cDNA of human EPOR was PCR amplified using primers flanking the cDNA and generating NotI and BamHI restriction sites. Following, the EPOR cDNA was cloned into the pMSCV-IRES-puromycin vector. The ΔC-mutant of CALR was generated by PCR using the primers CGGCAGGAATTCATGCTGCTATCCGTGCCGCTGCTG (EcoRI-CALR for) and CAGGACGAGGAGCAGAGGGACTACAAGGACGACG ACGATAAGTAAGCGGCCGCCATC (CALR-flag-stop NotI rev) and cloned the fragment into the pcDNA5/FRT/TO vector. The stop-codon was inserted after arginine (R) 366.

Reagents and Antibodies

The monoclonal rabbit anti-mouse/human GATA-1 (D52H6) antibody was ordered from Cell Signaling/New England Biolabs (NEB, Frankfurt, Germany). For the detection of YFP anti-GFP (600-103-215, Rockland, Gilbertsville, USA) was used for immunoblotting. Human TPO and EPO were purchased from ImmunoTools (Friesoythe, Germany).

RT-qPCR

The sequences of primers used for RT-qPCR were as follows: TTGTCGCACCTCAGTTACCT (mu Fli-1 for), TCTTGCCCATGGTCTGTGAT (mu Fli-1 rev), GAAGGTGAAGGTCGGAGT (hu Gapdh for), GAAGATGGTGATGGGATTTC (hu Gapdh rev), GTGCTGTTGTCACACCTCAG (hu Fli-1 for), TACTGATCGTTTGTGCCCCT (hu Fli-1 rev), TCGATCTCAAGCCGACTCTC (hu Ets-1 for), CATTCACAGCCCACATCACC (hu Ets-1 rev), GAAACTCTTCCTGCCCGTC (mu *EpoR* for), TGAGATGCCAGAATCGGACA (mu *EpoR* rev),

GATTGTCAGCAAACGGGCAG (mu *Gata-1* for), CGGTTCACCTGATGGAGCTT (mu *Gata-1* rev), TGCAGGAAGACAGTGGACAG (mu G-csfR for), GTGAAGAGGTCCCTGCTTTG (mu G-csfR rev). The mRNA expression level of the target gene is determined in % of *Gapdh* using 2^-ΔCT.

Native–PAGE

Cell lysates were prepared with RIPA buffer (see above) without sodium deoxycholate. Without prior boiling, lysates were electrophoresed on a non-denaturing (native) 8% polyacrylamide gel with cooling system. Western blotting was performed as described above.

Apoptosis assay

32D cells were seeded at a density of 5 x 10^5 cells/ml and were cultured in WEHI-free RPMI medium. Apoptotic cells were measured 48 h later by Annexin V-APC/7-aminoactinomycin D (7-AAD) (APC Annexin V Apoptosis Detection Kit, Biolegend, San Diego, CA, USA), using a Gallios flow cytometer (Beckman Coulter, Krefeld, Germany).

Transient transfection

HEK293T cells (4 x 10^6) were subcultured on a 10 cm dish and the day after transiently transfected with the vector pcDNA5/FRT/TO (2 µg) including the cDNA of WT CALR-flag, CALR del52-flag and CALR-ΔC-flag or empty vector (EV), respectively. The transfection reagent TranIT-LT1 (Mirus Bio LCC) was used as recommended by the instruction. After incubation of 24 h cellular lysates were prepared.
