# Supplementary figures and images for: Calreticulin-mutant proteins induce megakaryocytic signaling to transform hematopoietic cells and undergo accelerated degradation and Golgi-mediated secretion
Source: J Hematol Oncol. 2016 May 13;9:45. doi: 10.1186/s13045-016-0275-0 (PMC4894373; doi:10.1186/s13045-016-0275-0)

# Additional file 2: Figure S2

**a**

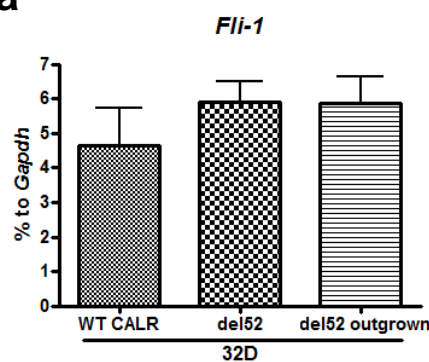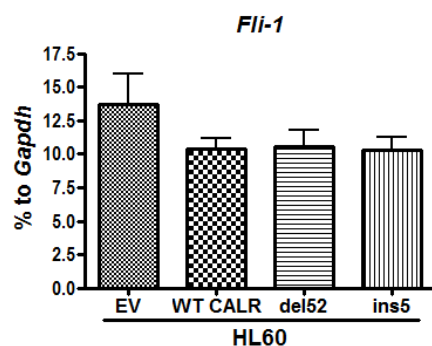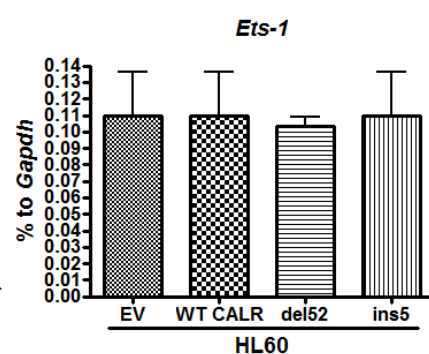

**b**

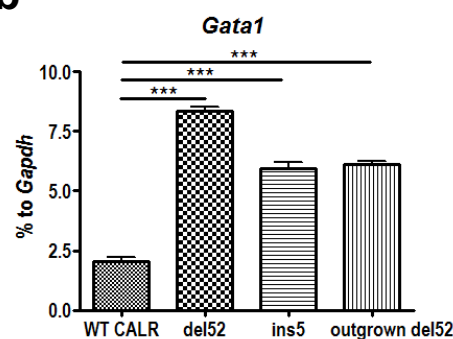

**c**

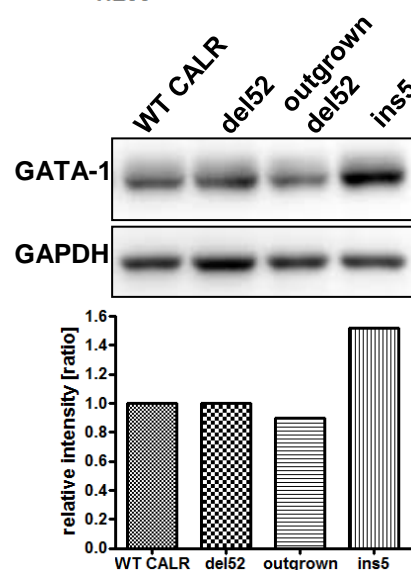

**d**

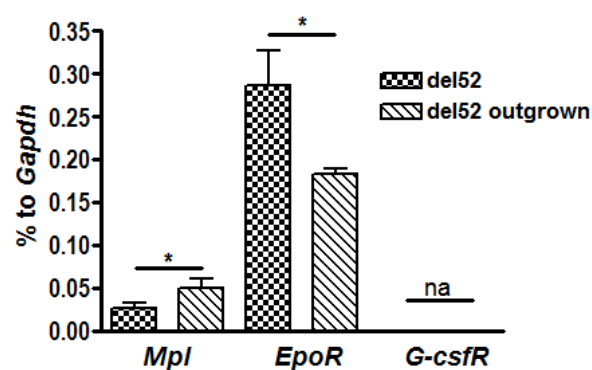

**e**

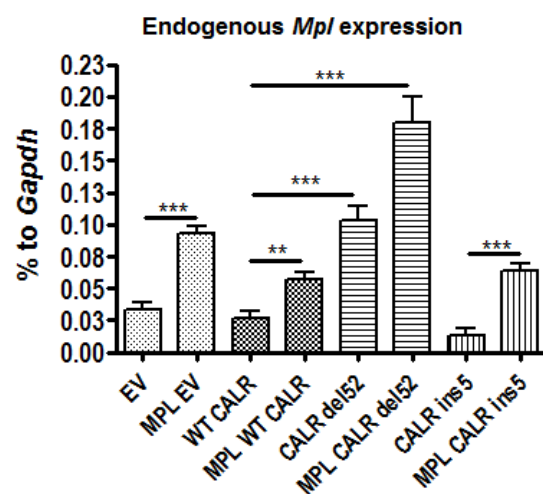

Supplement: Supplementary file 2 — Regulation of megakaryocytic factors by CALR mutants in murine and human cell lines. a Detection of Fli-1 and Ets-1 or b Gata1 mRNA expression by RT-qPCR in the indicated 32D and HL60e cells. The experiments were performed in triplicates. Mean and SD are indicated. ***P < 0.001. c 32D expressing WT CALR, CALR del52, CALR ins5 cells, and outgrown 32D del52 cells were used to prepare lysates. SDS-PAGE and Western blotting were performed. An antibody for the detection of GATA-1 protein was used for immunostaining. GAPDH served as loading control and was used for the calculation of GATA-1 expression ratios. d RT-qPCR was used to detect Mpl, EpoR, and G-csfR mRNA amounts after RNA isolation of the indicated 32D cells followed by cDNA synthesis. Expression is depicted in percentage to Gapdh. Measurements were done in triplicates. Mean and SD are indicated. *P < 0.05. e Analysis of endogenous Mpl expression by RT-qPCR to compare 32D EV, WT CALR, CALR del52, and CALR ins5 expressing cells +/− ectopic MPL expression. Experiments were performed in triplicates. Mean and SD are indicated. **P < 0.01, ***P < 0.001. (PDF 55 kb) [file 13045_2016_275_MOESM2_ESM.pdf]

### Additional file 3: Figure S3

**a**

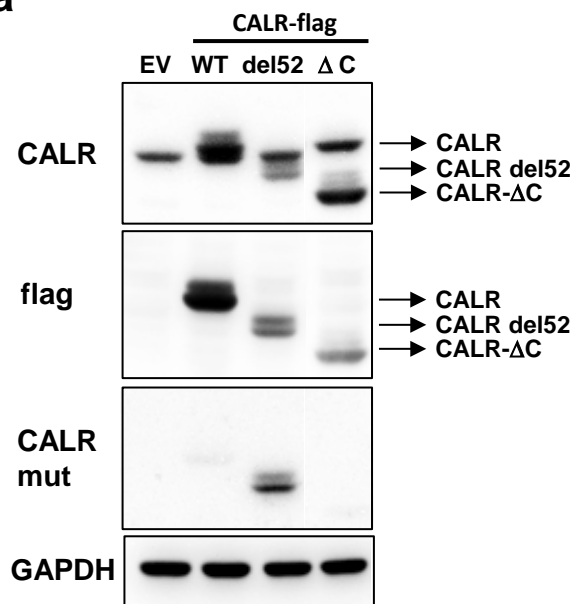

**b**

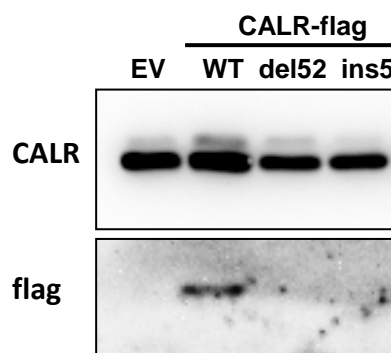

Supplement: Supplementary file 3 — Missing CALR-mutant detection in Western blotting is not due to loss of the antibody epitope or an SDS-PAGE artifact. a HEK293T cells were transiently transfected with expression vectors for flag-tagged WT CALR, CALR del52, a C-terminal truncation mutant (ΔC; aa R366) and empty vector. Twenty-four hours after, transfection lysates were generated and SDS-PAGE followed by Western blotting were performed. The indicated antibodies were used for immunostaining. The used CALR antibody bound to the CALR ΔC mutant. b Native page followed by Western blotting was performed with non-denatured protein lysates of 32D CALR cells. The PVDF membrane was stained with CALR antibody, and the membrane was stripped and stained with a flag-specific antibody. (PDF 67 kb) [file 13045_2016_275_MOESM3_ESM.pdf]

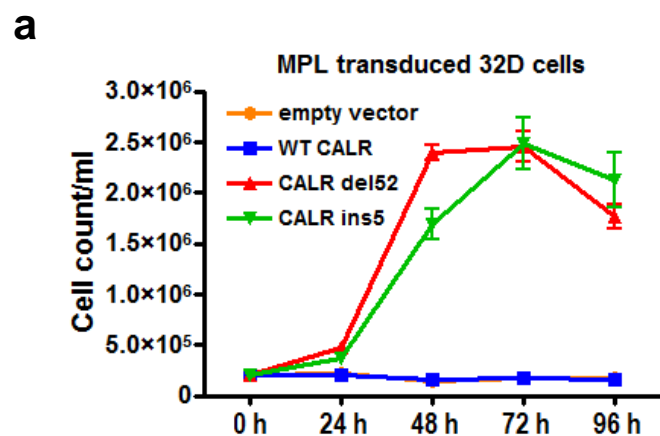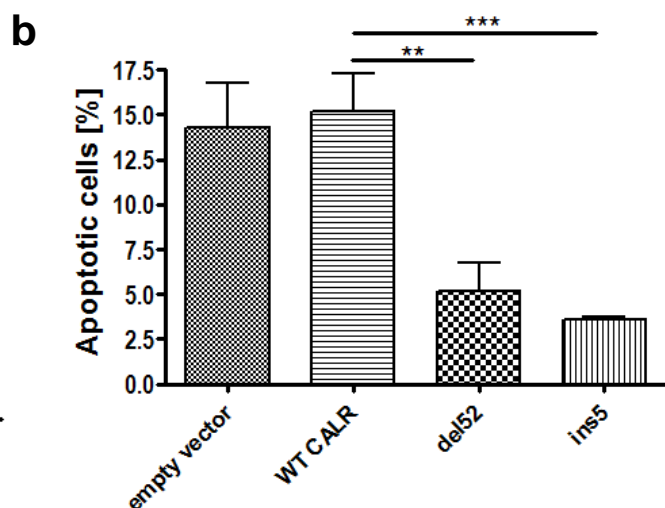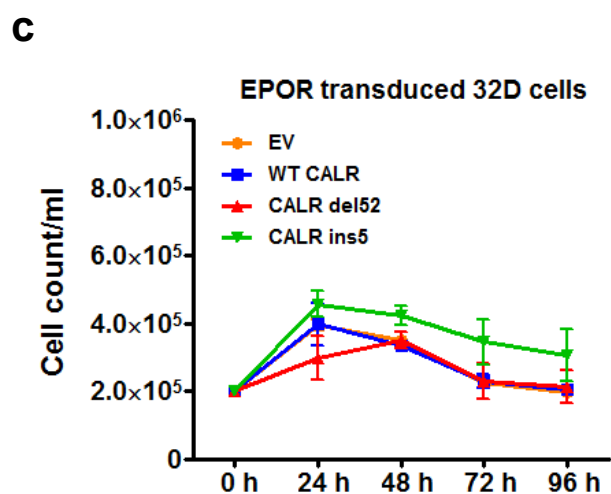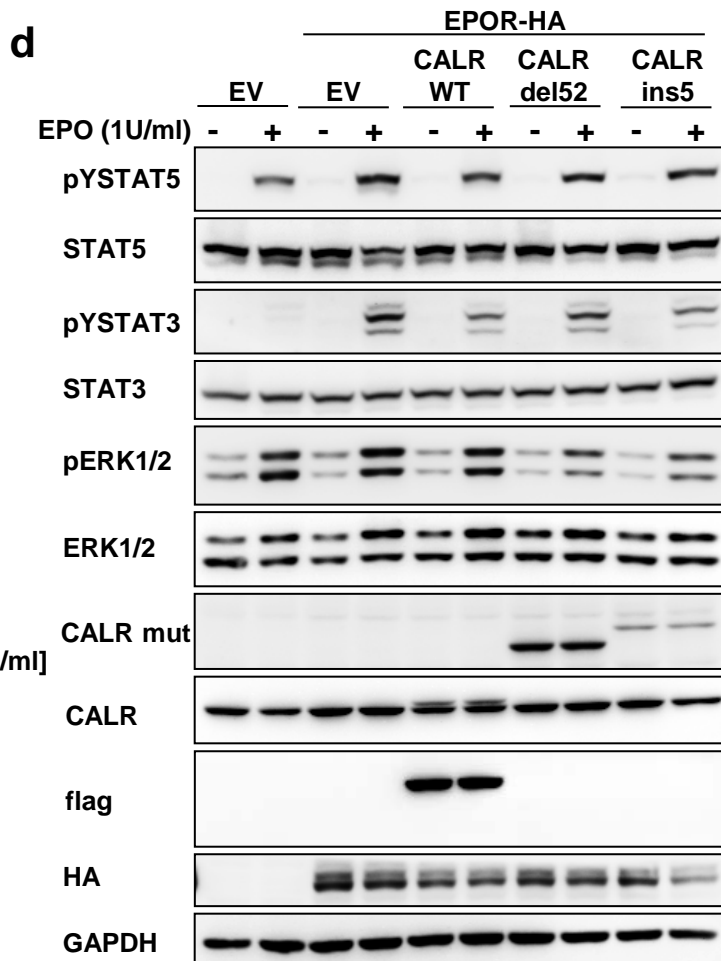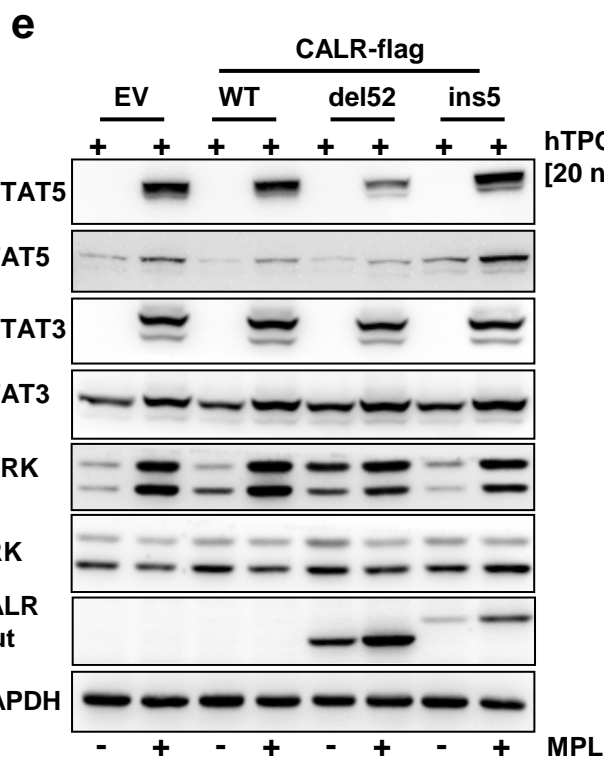

Supplement: Supplementary file 4 — Mutant CALR grants factor-independence, protects from apoptosis, and activates downstream signaling in an MPL-dependent manner. a A proliferation assay was performed with the indicated cell lines (2 × 10^5^ cells/ml). 32D MPL cells were counted every 24 h for 4 days. The cell counts are mean values of triplicates. b Empty vector, WT CALR, CALR del52, and ins5 expressing 32D cells were seeded in a density of 5 × 10^5^ cells/ml and grown for 48 h in WEHI-free medium. Apoptosis was analyzed by flow cytometry after staining with Annexin V-APC and 7-AAD. Mean and SD are indicated. *P < 0.05, **P < 0.01, ***P < 0.001. c The indicated 32D cell lines were stably transduced with the EPOR and a proliferation assay was performed (2 × 10^5^ cells/ml). The cells were counted every 24 h for 4 days. The cell counts are mean values of triplicates. d After 18 h starvation the indicated 32D cell lines were stimulated for 15 min with 1 U/ml EPO and lysates were prepared. SDS-PAGE, Western blotting and immunostaining with the indicated antibodies was performed. GAPDH served as loading control. e Stably transduced 32D cells with the indicated CALR-flag constructs +/− MPL were starved for 16 h and stimulated with 20 ng/ml human TPO for 15 min. Lysates were prepared and subjected to SDS-PAGE and immunoblotting using antibodies against phospho-STAT5, phospho-ERK1/2, phospho-STAT3, ERK1/2, STAT5, STAT3, and mutated CALR (CALR mut). GAPDH served as loading control. (PDF 172 kb) [file 13045_2016_275_MOESM4_ESM.pdf]

# Additional file 5: Figure S5

**a**

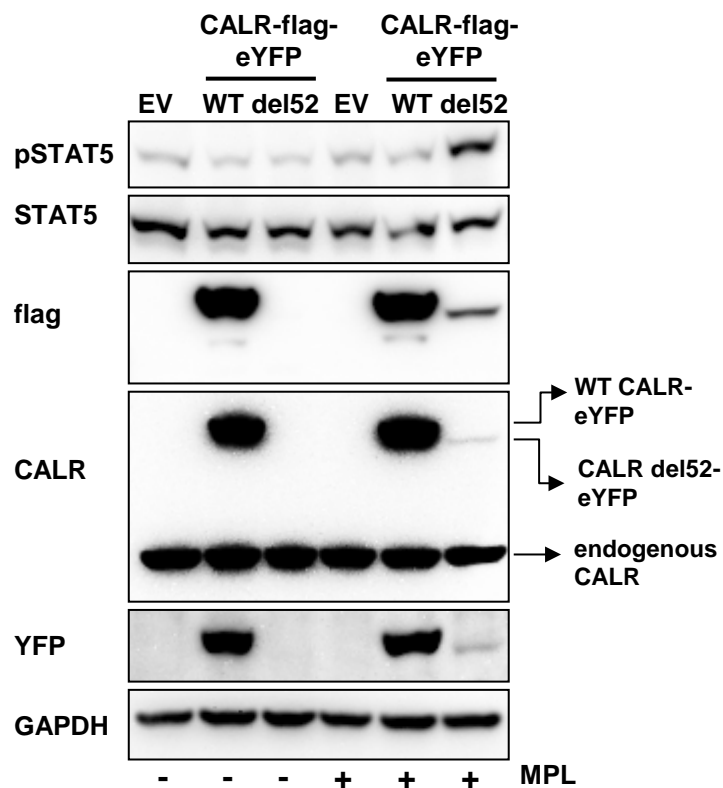

**b**

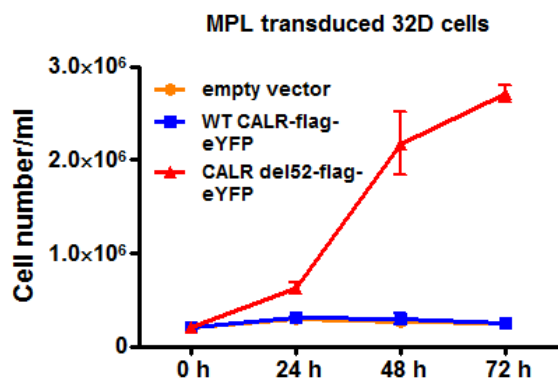

Supplement: Supplementary file 5 — Expression of the C-terminally YFP-tagged CALR del52 mutant leads to STAT5 phosphorylation and cytokine-independent growth of 32D MPL cells. a 32D cells stably transduced with WT CALR-flag-YFP, CALR del52-flag-YFP, or empty vector (EV) −/+ MPL were WEHI-starved for 16 h and protein lysates were prepared. SDS-PAGE and Western blotting was performed and the indicated antibodies were used for immunodetection. YFP was detected using a GFP-specific antibody, and GAPDH served as loading control. b The indicated 32D MPL cells were cultured in WEHI-free medium (2 × 10^5^ cells/ml). The cells were counted every 24 h for 3 days. The cell counts are mean values of triplicates. (PDF 53 kb) [file 13045_2016_275_MOESM5_ESM.pdf]
